# Haemophagocytic lymphohistiocytosis in patients treated with immune checkpoint inhibitors: analysis of WHO global database of individual case safety reports

**DOI:** 10.1186/s40425-019-0598-9

**Published:** 2019-05-02

**Authors:** Roberta Noseda, Raffaela Bertoli, Laura Müller, Alessandro Ceschi

**Affiliations:** 1 Division of Clinical Pharmacology and Toxicology, Institute of Pharmacological Sciences of Southern Switzerland, Ente Ospedaliero Cantonale, Via Tesserete 46, 6903 Lugano, Switzerland; 2 Department of Clinical Pharmacology and Toxicology, University Hospital Zurich, Zurich, Switzerland

**Keywords:** Immune checkpoint inhibitor, Haemophagocytic lymphohistiocytosis, VigiBase

## Abstract

**Background:**

Immune checkpoint inhibitor (ICI) use in clinical practice has unravelled a spectrum of immune-related adverse events (irAEs) due to immune system hyper-activation. ICI-related haemophagocytic lymphohistiocytosis (HLH) has been recently outlined in single case reports, raising a concern about the need of increasing our knowledge on this rare yet life threatening ICI haematological toxicity.

**Methods:**

To determine ICI-related HLH clinical, haematological, and coagulation features, its timing and outcome, concurrent irAEs and concomitant infections, we performed a retrospective observational cross-sectional study and queried VigiBase, the WHO global database of suspected adverse drug reactions (ADRs), on September 30th, 2018. We retrieved the individual case safety reports reporting HLH in association with ipilimumab, nivolumab, pembrolizumab, atezolizumab, avelumab or durvalumab, gathered in the database starting from the ICIs’ approval dates by the US Food and Drug Administration. The main outcome measures were co-suspected drugs, concurrent irAEs, HLH clinical, haematological and coagulation features, concomitant infections, HLH median time to onset and outcome.

**Results:**

Among 49′883 ICI-related ADRs collated in VigiBase as of September 30th, 2018, HLH was reported in 38 cases of which 34 (90%) mentioned ICIs as the solely suspected drugs. ICI-related HLH showed clinical, haematological and coagulation features similar to those of HLH with different etiology. Concurrent irAEs occurred in 5 (13%) patients and 6 (16%) reported concomitant viral infections. 31 (82%) cases defined ICI-related HLH outcome, which resolved in 19 (61%) cases. HLH developed a median of 6.7 weeks after initiation of ICI treatment (IQR 2.9–15.4, *n* = 18, 47%).

**Conclusions:**

By evaluating the largest cohort of ICI-related HLH cases, we observed that ICI-related HLH arises with a delayed timing with respect to initiation of ICI treatment, and usually presents without other irAEs and concomitant infections. Keeping in mind these findings, clinicians should consider ICIs’ involvement in the onset of HLH whenever they diagnose a disease of this group of syndromes in cancer patients treated with ICIs.

## Background

Haemophagocytic lymphohistiocytosis (HLH) is a rare life threatening group of syndromes characterised by hyper-activation of the immune system, which can lead to progressive organ damage and death [1]. According to the etiology, HLH divides into primary (genetic) and secondary (acquired). The latter is usually triggered by more than one external causative factors (e.g. infections and exposure to drugs with immunomodulatory effects) and underlying autoimmune diseases as well as neoplasms can increase the risk of HLH [2].

Nowadays, cancer immunotherapy with immune checkpoint inhibitors (ICIs) is progressively becoming a cornerstone in the treatment of a number of cancer types. However, by removing the normal inhibitory control that negatively regulates T-cell function, ICIs may result in T-cells hyper-activation and immune-related adverse events (irAEs) [3]. Therefore, ICI toxicity appears to partially overlap the dysregulated immune activation characterising HLH. In literature, information on HLH during treatment with ICIs is limited to a handful of case reports showing a broad range of symptoms, different therapeutic interventions and different outcomes [4–12]. We used VigiBase, the World Health Organization (WHO) global database of individual case safety reports [13] to describe the largest-to-date cohort of ICI-related HLH cases reported in clinical practice. Increasing knowledge on ICI-mediated HLH clinical presentation, timing, and outcome might facilitate the recognition of this multifaceted haematological toxicity, which could be erroneously attributed uniquely to the underlying cancer or other systemic inflammatory processes.

## Methods

VigiBase (http://www.vigiaccess.org/) is the largest pharmacovigilance database in the world that gathers spontaneously reported individual case safety reports of suspected adverse drug reactions (ADRs). The safety reports originate from over 130 member countries participating to the WHO Programme for International Drug Monitoring. Different types of reporters (e.g. physicians, pharmacists, other health care professionals, and patients) can contribute to generate safety reports, whereas a small proportion of reports derives from clinical studies. The scope of VigiBase is to identify novel ADRs and to gain knowledge on specific features of ADRs (e.g. spectrum, time to onset, and outcome) [13]. In VigiBase, reports are recorded in a structured form and include general administrative information (country of origin, reporting date, case seriousness), patient characteristics (age, sex), drugs (indication, start and end dates), and reactions (reported and coded term, onset date, outcome) details. The Medical Dictionary for Regulatory Activities (MedDRA - version 21.0 at the time of the study) allows for reactions’ coding into hierarchal groups. For this retrospective observational cross-sectional study, we selected cases reporting the MedDRA preferred term “histiocytosis haematophagic”, which comprised histiocytosis haemophagocytic, haemophagocytic lymphohistiocytosis, haemophagocytic syndrome, and macrophage activation syndrome. For clarity, throughout the manuscript, we used the term HLH to refer to this group of syndromes. We queried VigiBase on September 30th, 2018, for HLH reports associated with ipilimumab, nivolumab, pembrolizumab, atezolizumab, avelumab, and durvalumab, gathered in the database starting from the approval date of each substance by the United States Food and Drug Administration. We assessed co-suspected drugs, concurrent irAEs, HLH clinical, haematological and coagulation features, concomitant infections, HLH median time to onset and outcome.

To get further insights into patient comorbidities, we searched in PubMed for case reports of ICI-related HLH (applying the same inclusion criteria as in the VigiBase query), and subsequently selected those matching with the safety reports we retrieved from VigiBase. We used frequency and percentage to summarize categorical variables, and median and interquartile range (IQR) for continuous variables. Analyses were carried out by Microsoft Excel (2010, Microsoft Corporation, Washington, USA).

## Results

Among 49′883 ICI-related ADRs collated in VigiBase as of September 30th, 2018, HLH was reported in 38 cases. The highest reporting rate of ICI-related HLH occurred in France (0.4%), whereas the lowest in the United States of America (0.03%, Table 1). Table 2 resumes the baseline characteristics of the identified reports. 29 (76%) involved males and median patient age was 63 years (IQR 45–72 years). Melanoma was the most common cancer type (21, 55%), followed by lung cancer (5, 13% - non-small cell lung cancer, *n* = 4, adenocarcinoma of lung, *n* = 1). ICIs were the solely suspected drugs in 34 (90%) cases, whereas 4 (10%) patients reported as additional suspected drugs other antineoplastic agents (*n* = 2) and antibacterial agents (n = 2). Out of 38 cases of ICI-associated HLH, 22 (58%) received an anti-Programmed-Death-1/Programmed-Death-Ligand1 agent (PD-1/PD-L1). Regarding treatment duration, 4 (11%) patients received a single administration, 19 (50%) had a prolonged treatment (median duration 9.9 weeks, IQR 5.9–25.9 weeks), and in 15 (39%) patients treatment duration could not be defined. ICI-mediated HLH developed a median of 6.7 weeks after initiation of ICI treatment (IQR 2.9–15.4 weeks, *n* = 18, 47%), and HLH reporting in association with ICIs increased over time (18 cases, 47%, in 2018, at the time of writing).

**Table 1:** Geographical pattern of immune checkpoint inhibitor-related haemophagocytic lymphohistiocytosis reporting rate

Country of primary source	Total number of ICI-related safety reports	Number of ICI-related HLH safety reports	ICI-related HLH reporting rate (%)
France	3526	14	0.4
Japan	6421	11	0.2
Germany	1901	3	0.2
Switzerland	830	1	0.1
Canada	1279	1	0.08
US of America	24'998	8	0.03

Abbreviations: *ICI* immune checkpoint inhibitor, *HLH* haemophagocytic lymphohistiocytosis

**Table 2:** Baseline characteristics of the individual case safety reports concerning immune checkpoint inhibitor-related haemophagocytic lymphohistiocytosis

Characteristic	Patients No. (%) (*n* = 38)
Age	
Reported	33 (87)
Median [IQR], years	63 [45–72]
Not reported	5 (13)
Sex	
Male	29 (76)
Female	9 (24)
Cancer type	
Melanoma	21 (55)
Lung cancer	5 (13)
Bladder cancer	3 (8)
Renal cell carcinoma	2 (5)
Hodgkin disease	1 (3)
Transitional cell carcinoma	1 (3)
Adenocarcinoma gastric	1 (3)
Thymoma	1 (3)
T cell lymphoblastic leukemia acute	1 (3)
Not reported	2 (5)
Co-suspected drugs	
Reported	4 (10)
Antineoplastic agents	2 (5)
Antibacterial agents	2 (5)
Not reported	34 (90)
Drugs	
Anti-CTLA-4 (ipilimumab) monotherapy	7 (18)
Anti-PD-1 monotherapy	
nivolumab	14 (37)
pembrolizumab	7 (18)
Anti-PD-L1 monotherapy	
Atezolizumab	1 (3)
ipilimumab and nivolumab combination therapy	5 (13)
nivolumab and ipilimumab sequential therapy	3 (8)
pembrolizumab and ipilimumab sequential therapy	1 (3)
Reporting	
2014	1 (3)
2015	3 (8)
2016	6 (16)
2017	10 (26)
2018	18 (47)

Abbreviations: *IQR* interquartile range, *CTLA-4* Cytotoxic T-Lymphocyte Antigen 4, *PD-1* Programmed cell Death protein 1, *PD-L1* Programmed cell Death-Ligand 1

All cases of ICI-related HLH were evaluated by reporters as serious for causing or prolonging hospitalization (16, 42%), for determining life threatening conditions (7, 18%), or because related to death (10, 26%). Five (13%) cases did not specify the seriousness criteria. Among the ten fatal cases, 4 (40%) mentioned HLH as the unique cause of death. In three (30%) patients, HLH contributed to death along with malignant neoplasm progression, multi-organ failure, and brain haemorrhage at the cerebral metastasis site, respectively. In the remaining 3 (30%) cases reporting death, HLH probably did not contribute to death which occurred either for sepsis (*n* = 1) or malignant neoplasm progression (*n* = 2). Besides fatal cases, HLH was not resolved at the time of reporting in 5 (16%) patients whereas recovered in 19 (61%) patients (out of 31 cases defining HLH outcome).

As shown in Table 3, concurrent irAEs occurred in 5 (13%) patients and HLH clinical, haematological and coagulation features were mentioned in 15 (%) cases. Concomitant viral infections were reported in 6 (16%) cases (Herpes zoster, *n* = 1; Epstein-Barr virus, *n* = 5).

**Table 3:** Haemophagocytic lymphohistiocytosis features and immune-related adverse events reported in patients treated with immune checkpoint inhibitors

Clinical features of HLH	Patients No. (%) ^a^ (n = 38)
Pyrexia	2 (5)
Pulmonary involvement	
Cough	1 (3)
Neurological involvement	
Encephalopathy	1 (3)
Headache	1 (3)
Psychiatric changes	
Delirium	1 (3)
Cutaneous involvement	
Generalised erythema	1 (3)
Drug eruption	1 (3)
Gastrointestinal involvement	
Enterocolitis	1 (3)
Diarrhoea	2 (5)
Renal involvement	
Renal failure	1 (3)
Renal tubular necrosis	1 (3)
Haematological and coagulation features of HLH	
Anaemia	1 (3)
Thrombocytopenia	2 (5)
Leukopenia/ White blood cell count decreased	2 (5)
Neutrophil count decreased	1 (3)
Disseminated intravascular coagulation	3 (8)
International normalised ratio abnormal	1 (3)
Pancytopenia/ Bone marrow failure	2 (5)
Concurrent irAEs	
Autoimmune hepatitis	2 (5)
Interstitial lung disease	2 (5)
Myositis	1 (3)
Thyroiditis	1 (3)
Cardiac^b^	1 (3)

Abbreviations: *HLH* haemophagocytic lymphohistiocytosis, *irAEs* immune-related adverse events

^a^ Some patients reported more than one adverse drug reaction besides HLH

^b^ Atrial fibrillation and left ventricular failure

When we searched the literature for case reports of ICI-associated HLH, we identified six cases matching with individual case safety reports retrieved from VigiBase. However, none of these case reports allowed gaining information on patients’ comorbidities.

## Discussion

We reported the largest-to-date analysis of ICI-associated HLH cases collated in the WHO global database of suspected ADRs. By blocking the signalling pathway that negatively regulates T cell activation, ICIs enhance the immune response against cancer cells and, aberrantly, against self-antigens triggering immune-related adverse events [3]. Hyper-activation of lymphocytes, natural killer (NK) cells and histiocytes are distinguishing features of HLH [1]. To date, a causative role for activated T cells in HLH development has been hypothesized in cancer patients as an additive effect to the excessive cytokine secretion induced by cancer cells [2, 14].

Remarkably, VigiBase does not provide data on the total number of patients treated with ICIs in clinical practice; therefore, the absolute incidence of HLH from ICI usage cannot be evaluated with such a source of data. Conversely, by gathering spontaneous safety reports from more than 130 countries, VigiBase allows for the assessment of the proportion of safety reports for a specific drug toxicity (i.e. ICI-related HLH) out of the total number of safety reports present in the database for the drug of interest (i.e. all ICI-related ADRs). In light of this evidence, we could observe that HLH was included in less than 0.1% of all safety reports associated with ICIs overall, thus confirming that it is a rare haematological toxicity upon treatment with ICIs.

We observed geographical variability of ICI-related HLH reporting rate across countries of primary source; in particular, we found that France, Germany and Japan had the highest ICI-related HLH reporting rates whereas the US had the lowest. This might suggest a genetic predisposition towards ICI haematological toxicity and HLH development, similarly to previous observations relating specific genetic backgrounds to different patients’ predisposition towards HLH triggering agents [2]. Moreover, we found that ICI-related HLH safety reports mostly involved melanoma patients, probably due to the earlier approval of ipilimumab and anti-PD-1 agents for melanoma, which, consistently, were the substances most commonly reported as suspected by the majority of ICI-related HLH safety reports.

The reporting of HLH as ICI-mediated toxicity is increasing over years. Although this likely depends on the progressively increasing use of ICIs across different cancer types, our study strengthens awareness of a novel and relevant pharmacological trigger for HLH, in addition to neoplasms and infections as well-known HLH predisposing factors [2]. Indeed, the majority of study cases mentioned neither underlying malignancies nor concomitant infections as HLH contributory causes besides ICIs. However, these findings must be adequately weighted when interpreted as, relying on spontaneous reporting, individual case safety reports are rarely sufficient to confirm that a specific drug caused the adverse event [13], and do not rule out the existence of other contributing causes of the reported ADR, e.g. the disease being treated, a new disease, other ADRs of the same drug, or other suspected drugs. Remarkably, the latter were mostly absent from the cases we analysed, suggesting ICIs pivotal role as contributing pharmacological trigger in the genesis of HLH.

Although the quality of the large amount of data stored in VigiBase is variable in that the reported information derives from different sources, with different levels of details and sometimes partial or missing data, VigiBase represents a unique source of information, allowing for the detection of the rarer and less frequently reported ADRs [13]. By exploiting this database, we managed to identify 38 cases of ICI-related HLH, far beyond the amount of cases published to date in the literature [4–12].

Since patient comorbidities are sparsely reported in VigiBase, with co-suspected and concomitant drugs providing limited insights, to try to overcome this limitation, we also reviewed published single case reports [4–12] that matched with the safety reports retrieved from VigiBase. Unfortunately, the information retrieved from these case reports was insufficient to identify predisposing conditions shared among patients developing HLH on ICI treatment.

HLH as adverse effect of drugs has been rarely reported in literature and mainly described in association with the systemic inflammatory response induced by drug reaction with eosinophilia and systemic symptoms (DRESS) syndrome [15], or as a consequence of an infection secondary to immunosuppressant agents [2]. More recently, antiepileptic drugs were associated with HLH onset, because of their immune-modulating action [16, 17]. Noteworthy, anticonvulsant drug-related HLH developed 2–3 weeks after treatment initiation, a time to onset relatively shorter if compared with the delayed time to onset that we observed with ICIs.

Confirming that HLH is a life threatening condition, reporters evaluated all ICI-related HLH cases as serious, although HLH resolved in the majority of cases, in agreement with the notion that this group of syndromes generally responds to systemic corticosteroids [2].

## Conclusions

In clinical practice, ICI-related HLH arises with a delayed timing with respect to initiation of ICI treatment, presents clinical, haematological and coagulation features similar to those of HLH with different etiology, rarely is associated with other irAEs and concomitant viral infections, and resolved in the majority of cases. Keeping in mind these findings, clinicians should consider ICIs’ involvement in the onset of HLH whenever they diagnose a disease of this group of syndromes in cancer patients treated with ICIs.

## Abbreviations

ADR: Adverse Drug Reaction
DRESS: Drug Reaction with Eosinophilia and Systemic Symptoms
HLH: Haemophagocytic lymphohistiocytosis
ICI: Immune Checkpoint Inhibitor
IQR: Interquartile Range
irAE: Immune-related Adverse Event
MedDRA: Medical Dictionary for Regulatory Activities
WHO: World Health Organization
